# Left main renal artery entrapment by diaphragmatic crura: spiral CT angiography

**DOI:** 10.2349/biij.6.2.e11

**Published:** 2010-04-01

**Authors:** S Singham, P Murugasu, J MacIntosh, P Murugasu, A Deshpande

**Affiliations:** Department of Medical Imaging, John Hunter Hospital, Newcastle, Australia

**Keywords:** renal artery stenosis, spiral computed tomography angiography

## Abstract

Entrapment of renal artery by the diaphragmatic crus is a rare cause of renal artery stenosis. Spiral computed tomography angiography provides a definitive diagnosis and shows the precise relationship of the artery to the diaphragmatic crus. The authors present a case of hypertension developing in a young 20-year-old female due to entrapment of the left renal artery by the diaphragmatic crus. This condition should be considered in young hypertensive patients with renal artery stenosis without cardiovascular risk factors.

## CASE REPORT

A 20-year-old female presented with shortness of breath on modest exertion. She described several recent episodes of lower retrosternal chest pain at rest. She denied any other symptoms. She had no other significant medical history but was a moderate-to-heavy smoker. There was no relevant family history.

Physical examination was normal. Full blood count and urea, creatinine and electrolyte levels were normal. Serial troponin levels were also normal. Chest x-ray was normal. Electrocardiography (ECG) showed no evidence of ischaemic change but, instead, a hypertensive response. She was noted to become hypertensive on walking up a flight of stairs. Her resting blood pressure was 130/70 mmHg and this increased to 200/100 mmHg on walking up a flight of stairs. Biochemical investigations she underwent, including tests for secondary causes of hypertension such as hyperaldosteronism and pheochromocytoma, renin aldosterone ratio as well as urinary biogenic amine, were normal. Lipid studies, thyroid function tests, liver function tests, coagulation screening, blood glucose level and HbA_1c_ were normal.

She was initially treated with perindopril and indapamide to control her blood pressure and the treatment was effective.

A renal Doppler ultrasound suggested the presence of an accessory left renal artery. A spiral computed tomography angiography (spiral CTA) was performed with axial and sagittal slab MIPs, curved planar reformation, and 3D MIPs.

The scan was performed using a Philips Brilliance 16-slice CT scanner with 500 ml excursion 120 kV 30 mA per second. Surview image was first obtained to locate the kidneys then a helical acquisition to include a centimetre above and a centimetre below the upper and lower poles of the kidneys, respectively, with 1 mm slices and 0.5 mm spaces in between. These images were reconstructed to 2 mm by 2 mm axial and coronal images. Contrast was injected at the rate of 4 ml per second using 75 ml of Ultravist 370.

**Figure 1:**
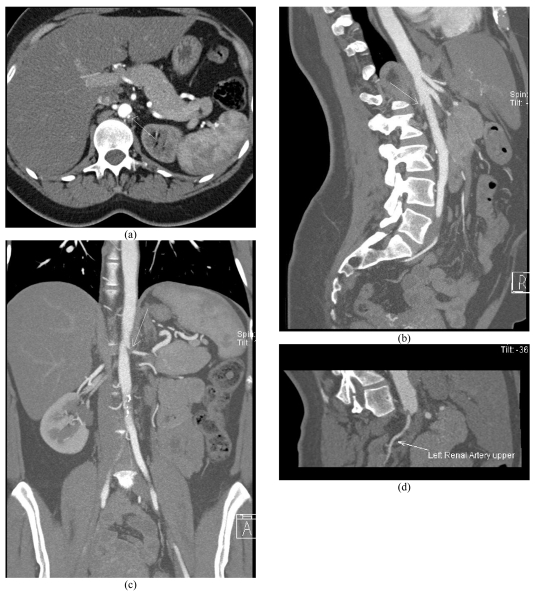
(a) Axial, (b) sagittal and (c) coronal slab MIP and (d) curved planar reformatted images. The left main renal artery has marked extrinsic compression with haemodynamically significant stenosis due to an aberrant and posterior course through the left crus of the diaphragm (arrows).

**Figure 2:**
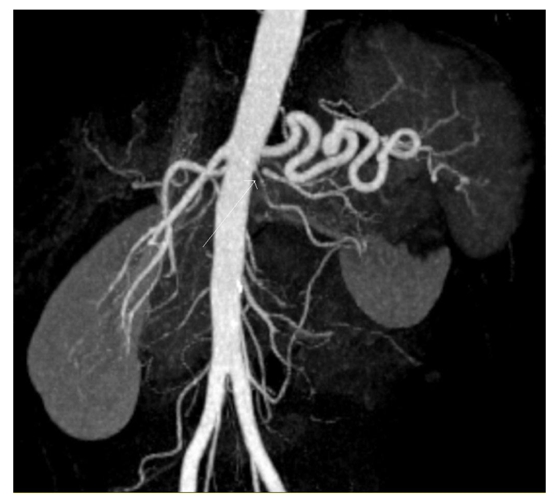
Coronal slab MIP. The lower pole of the left kidney is supplied by an accessory small calibre renal artery (white long arrow).

**Figure 3:**
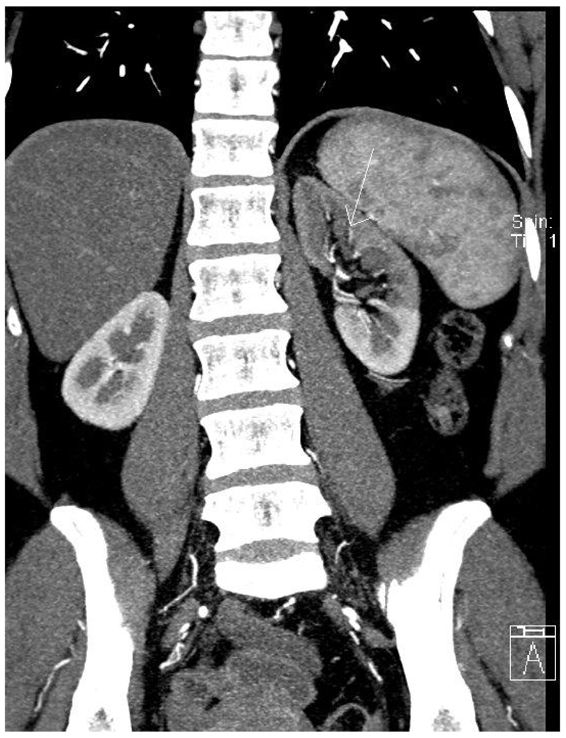
Coronal image demonstrates decreased perfusion to the upper pole and mid left kidney (white arrow). There is normal perfusion to the left lower pole and right kidney.

Review of the images demonstrates two left renal arteries and a single right renal artery. The right renal artery has a normal origin and normal calibre throughout its course. The left main renal artery supplies the upper pole and the mid renal parenchyma. It has a more posterior origin and marked extrinsic compression producing a haemodynamically significant stenosis as it passes aberrantly through the left crus of the diaphragm (Figure 1a, 1b, 1c, 1d). The left lower pole renal artery is smaller in calibre with a normal course (Figure 2). There was decreased perfusion to the left upper pole and mid renal parenchyma (Figure 3).

**Figure 4:**
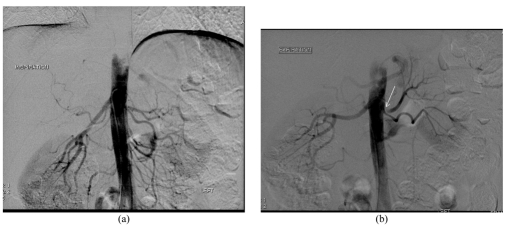
Abdominal angiogram in (a) inspiration, and (b) expiration. Images demonstrate a single right normal renal artery and a normal left lower pole renal artery. The left main renal artery to the upper pole of the left kidney was moderately narrowed (white arrow) at the origin with mild post stenotic dilation.

Subsequently, an aortogram with bilateral renal artery selection was performed during suspended inspiration and expiration (Figure 4a and 4b). The upper pole renal artery (main) is confirmed to arise posterior and superior to the lower pole renal artery with a sharp downward bend. There was moderate narrowing at its origin with mild post stenotic dilatation, seen best on the inspiratory acquisition (Figure 4a and 4b). Selective angiography of the upper pole artery was attempted, but the catheter could not be passed due to sharp angulation at the origin. There was no evidence of pre-existing stenosis, atherosclerosis or fibromuscular dysplasia of the left lower pole renal artery.

Unfortunately, despite best attempts, this patient was lost to follow up.

## DISCUSSION

Renal artery entrapment by the diaphragmatic crus was first described by D’Abreu [1] who reported two cases proven by surgery in 1962. Since this first description, less than 20 cases have been reported in the literature. Congenital abnormalities such as abnormal musculo-tendinous fibres, high ectopic renal artery origin or hypertrophic diaphragmatic crus were found to be responsible for these entrapments [2].

Renal artery stenosis (RAS) is a common, correctable cause of hypertension and renal impairment. In the general hypertensive population the prevalence of this condition varies between 1 and 5%. The most common causes of RAS are atherosclerosis and fibromuscular dysplasia [3].

Extrinsic compression of the renal arteries leading to hypertension has been associated with abdominal aortic aneurysm [4], tumour, hypertrophic adrenal tissue, and psoas muscle band anomaly [5]. However, extrinsic compression of one or both renal arteries by the diaphragmatic crura, which is known as renal entrapment syndrome, is rare [6-10]. Compression is by fibres forming part of the crus of the diaphragm or psoas muscle impinging on the renal artery by verticalisation of the root of the renal artery. This results in stenosis (usually at the ostium of the artery). The artery follows an unusual acutely angled (sigmoid) course. This anomaly is also associated with a high origin of the renal artery from the aorta and is more common on the left side. The mechanism evoked is an anomaly of migration of the kidneys [11].

Clinical features suggestive of RAS include abdominal bruit, severe retinopathy, unexplained hypokalaemia, and unexplained renal impairment [3]. Early detection of RAS is necessary for effective treatment and to prevent end-stage renal disease [12].

Renal artery entrapment may be suspected on angiographic views and proven by cross-sectional imaging [2]. Thony et al. [2] demonstrated two angiographic features suggesting renal artery entrapment: renal arteries descending down and close to the aorta, and a concentric ostial stenosis in a patient free of atheroma.

Although Duplex ultrasound is an accurate examination for screening RAS [13], it does not allow the analysis of the relationship between the renal artery and muscular structures [2]. This is clearly shown in angiographic reconstructions using CT [2].

Although surgery and stenting have been used for treatment of renal entrapment syndrome, they are associated with surgical morbidity and stent-related complications such as bending or rupture of stents. Surgical treatment needs to be considered on a case-by-case basis in relation to the anatomy and the biological and functional data. The use of an arterial stent in the situation of muscular compression leads to a risk of bending or rupture of the stent [11]. In addition, movement of the diaphragm induces significant displacement of kidneys during respiration which induces both bending and torsional forces on the renal arteries. This bending may lead to stent fracture and restenosis [14]. An alternative is to treat with balloon angioplasty and cutting balloon angioplasty, which may have lower patency rate but fewer stent-related complications in these patients [15].

Bilici et al. [16] have investigated the use of botulinum toxin injection directly into the diaphragmatic crus under CT guidance as an alternative to surgical treatment and stenting. This method still requires further evaluation.

## CONCLUSION

Compression of a renal artery by the crus of the diaphragm (renal entrapment syndrome) should be investigated in proximal renal artery stenosis in young hypertensive patients without other cardiovascular risk factors, and where fibromuscular dysplasia is unlikely. Spiral CTA is a key investigation for identification of the renal entrapment syndrome. Once the renal entrapment syndrome is confirmed, surgical management should be a consideration. New treatment methods are being evaluated including botulinum injection, which may provide an alternative to surgical management.

## References

[1] d' Abreu, Strickland B (1962). Developmental renal-artery stenosis. Lancet.

[2] Thony F, Baguet JP, Rodiere M (2005). Renal artery entrapment by the diaphragmatic crus. Eur Radiol.

[3] Paven G, Waugh R, Nicholson J (2006). Screening tests for renal artery stenosis: a case-series from an Australian tertiary referral centre. Nephrology (Carlton).

[4] Lepke RA, Pagani JJ (1982). Renal artery compression by an aortic aneurysm: an unusual cause of hypertension. AJR Am J Roentgenol.

[5] Lampe WT (1965). Renovascular hypertension. A review of reversible causes due to extrinsic pressure on the renal artery and report of three unusual cases. Angiology.

[6] Vahdat O, Creemers E, Limet R (1991). [Stenosis of the right renal artery caused by the crura of the diaphragm. Report of a case]. J Mal Vasc.

[7] Bacourt F, Depondt JL, Lacombe P (1992). [Compression of the left renal artery by the diaphragm]. J Mal Vasc.

[8] Clement C, Ruiz R, Costa-Foru B (1990). Extrinsic compression of the renal artery by diaphragmatic crus. Ann Vasc Surg.

[9] Deglise S, Corpataux JM, Haller C (2007). Bilateral renal artery entrapment by diaphragmatic crura: a rare cause of renovascular hypertension with a specific management. J Comput Assist Tomogr.

[10] Dure-Smith P, Bloch RD, Fymat AL (1998). Renal artery entrapment by the diaphragmatic crus revealed by helical CT angiography. AJR Am J Roentgenol.

[11] Baguet JP, Thony F, Sessa C (2003). Stenting of a renal artery compressed by the diaphragm. J Hum Hypertens.

[12] Glockner JF, Vrtiska TJ (2007). Renal MR and CT angiography: current concepts. Abdom Imaging.

[13] Nchimi A, Biquet JF, Brisbois D (2003). Duplex ultrasound as first-line screening test for patients suspected of renal artery stenosis: prospective evaluation in high-risk group. Eur Radiol.

[14] Draney MT, Zarins CK, Taylor CA (2005). Three-dimensional analysis of renal artery bending motion during respiration. J Endovasc Ther.

[15] Chua SK, Hung HF (2009). Renal artery stent fracture with refractory hypertension: a case report and review of the literature. Catheter Cardiovasc Interv.

[16] Bilici A, Karcaaltincaba M, Ilica AT (2007). Treatment of hypertension from renal artery entrapment by percutaneous CT-guided botulinum toxin injection into diaphragmatic crus as alternative to surgery and stenting. AJR Am J Roentgenol.

